# A Robust Variable Sampling Time BLDC Motor Control Design Based upon *μ*-Synthesis

**DOI:** 10.1155/2013/236404

**Published:** 2013-11-12

**Authors:** Chung-Wen Hung, Jia-Yush Yen

**Affiliations:** ^1^Department of Electrical Engineering, National Yunlin University of Science and Technology, Yunlin, Douliou 64002, Taiwan; ^2^Department of Mechanical Engineering, National Taiwan University, Taipei 10617, Taiwan

## Abstract

The variable sampling rate system is encountered in many applications. When the speed information is derived from the position marks along the trajectory, one would have a speed dependent sampling rate system. The conventional fixed or multisampling rate system theory may not work in these cases because the system dynamics include the uncertainties which resulted from the variable sampling rate. This paper derived a convenient expression for the speed dependent sampling rate system. The varying sampling rate effect is then translated into multiplicative uncertainties to the system. The design then uses the popular *μ*-synthesis process to achieve a robust performance controller design. The implementation on a BLDC motor demonstrates the effectiveness of the design approach.

## 1. Introduction

The brushless DC (BLDC) motor applications are getting popular due to their efficient operation and high power density characteristics. Because of its high efficiency and easy maintenance, the application range has been found from large system like electrical vehicles to small systems like computer peripherals. The BLDC motor usually uses hall-effect sensors to determine the commutation timing among the armature windings. The same hall sensor signal is also used to measure motor speed. There are many commercial industrial driver ICs which apply such kind of design.

BLDC motors may be standard application, but ones still encountered many difficulties when engineers try to implement in precision speed control. As mentioned before, the typical BLDC motor feedback signal is from the few hall-effect sensor signals which originally are designed for commutation purpose. The time interval between the consecutive feedback signals depends on the motor speed. Notice that BLDC motor is used in many high performance industrial applications like computer hard disk drive and the optical drive servosystems. High performance controller design is necessary. The BLDC motor system design maintains high resolution speed measurement through high frequency clock. The traditional driver design uses current transducer to achieve constant current sampling for the acceleration loop; however, the velocity loop has to be based on the speed dependent sampling, and some sort of robust variable sampling rate controller design is thus desirable.

Most previous researches on multirate sampling systems in the literatures have focused on multiple but fixed sampling rate problems [1–6]. If one examines the system with the slow sampling rate, the multirate control offers improved performance. Some people have also applied multirate control to achieve smoother system responses due to the fact that controls can be updated more frequently than the measurements [7, 8]. Moore et al. [9] summarized these control strategies into an N-delay input/output control and have demonstrated successful implementation [10]. Even though the intervals in this approach do not need to be uniform, the result still requires a fixed “N” step delay. These literatures did not focus on variable sampling system. The unpredicted sampling period really imposes a barrier on the theoretical development. In 1993, Hori published an interesting result in [11], which he considered a pure integrator system, and was able to reduce the speed-position relation into a (time) invariant system (thus, guaranteed error convergence). This result is only valid for pure integrator, but there is still a long way to a more complete result. However, an accurate system model could be derived, and the controller should be able to draw information from it. The studies in [12, 13] confirm that such approaches may be beneficial. References [14, 15] discussed the variable sampling status caused by networked-induced variable delay and developed the controller. But the delay is limited in some interval range. Reference [16] implemented the neural network to compensate the delay caused by networked control systems. References [17, 18] used fuzzy control to handle the variable sampling system.

In the preceding work [19], the authors derived the variable sampling frequency system model for the observer design and used singular value assignment to guarantee the convergence of the observation error. A model based compensator is designed and then forms the control bases. The approach is basically systematic, and because the BLDC motor system is of low order in general, the singular value assignment procedure does not run into too much difficulty. The singular value assignment process is inherently very conservative. From our experience it does not always provide robust observation. Plus, there is no access to the stability of the feedback system. References [20, 21] proposed a series of methods to simulate the variable sampling system and predict cheaply the results without hardware experiments. Reference [22] designed an adaptive antiwindup PID sliding mode controller for the EHA system. The experimental showed that the controller could against hardware saturation, load disturbance, and lumped system uncertainties and nonlinearities. But it did not discuss variable sampling system. In [23], the authors improved the discrete-time variable structure control and modified the fixed system time into variable sampling time. It could successfully provide stable and fast response; however the controller took too much MCU calculation resource and is finally implemented in look-up table method. 

In this paper, the authors realized that the variable sampling rate system model derived for the observer can be reduced to describe the variable sampling rate system itself. Instead of treating the system with a prespecified sampling rate, one can look at the system only when the feedback is available and lump the effects of the variable sampling time into system uncertainties. It is now possible to describe the system with the standard linear fractional transformation. The *μ*-synthesis can now provide a necessary and sufficient condition for the controller design. The simulation and the experimental results demonstrate very satisfactory results from this method.

## 2. Problem Formulation

**Figure 1:**
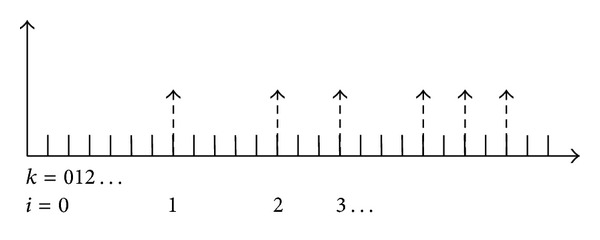
The variable rate time frames.

Consider two time scales, the underlying system sampling rate is denoted by *k*, and the other one describing the measurement update is denoted by *i*. Therefore, as mentioned above, the *i* sampling system has a variable sampling rate.  Figure 1 illustrates the relation between the two time frames.

If the basic sampling time for the system is Δ*t*, the system could be described as

(1) $$\begin{array}{c} x\left(k+1\right)=Ax\left(k\right)+Bu\left(k\right),\\ y\left(k\right)=Cx\left(k\right), \end{array}$$

where *A* = *e* ^*F*Δ*t*^, *B* = ∫_0_^Δ*t*^ *e* ^*Fτ*^ *Gdτ*, *F* ∈ *ℜ* ^*n*×*n*^ is the continuous-time system matrix, *G* ∈ *ℜ* ^*n*×1^ is the continuous-time input matrix, *n* is the system order, and *C* ∈ *ℜ* ^1×*n*^ is the same in the discrete-time system as in the continuous-time system.

For the design purpose, assume that the system model is precisely known, and the manipulated input *u*(*k*) is known at every instance. This is a valid assumption because the system model can be measured with various system identification processes, and the control input is from the known controller. 

Consider that “*k*_*i*_” stands for the instance when the *i*th measurement is available. “*m*_*i*_ = *k*_*i*+1_ − *k*_*i*_” stands for the number of fast samples from the instance “*i*” to the instance “*i* + 1.” The control law is updated only when the measurement is available; therefore the system description becomes the following equations:

(2) x(k+1)=Ax(k)+Bu(i), for  ki≤k<ki+1,x(ki+1)=Ax(ki)+Bu(i),x(ki+2)=Ax(ki+1)+Bu(i)=A[Ax(ki)+Bu(i)]+Bu(i)=A2x(ki)+ABu(i)+Bu(i),

and so forth.

Thus, for every instance *i*, (3) could be derived

(3) $$\begin{array}{c} x\left(k_{i+1}\right)=A^{m_{i}}x\left(k_{i}\right)+\sum_{j=0}^{m_{i}-1}A^{m_{i}-j-1}Bu\left(k_{i}+j\right). \end{array}$$

Notice that *u*(*k*_*i*_ + *j*) = *u*(*i*), for *k*_*i*_ ≤ *k* < *k*_*i*+1_ in our case; if one considers the (*i* + 1)th measurement instance, then the system becomes

(4) $$\begin{array}{c} x\left(i+1\right)=A^{m_{i}}x\left(i\right)+\sum_{j=0}^{m_{i}-1}A^{m_{i}-j-1}Bu\left(i\right),\\ y\left(i\right)=Cx\left(i\right). \end{array}$$

**Figure 2:**
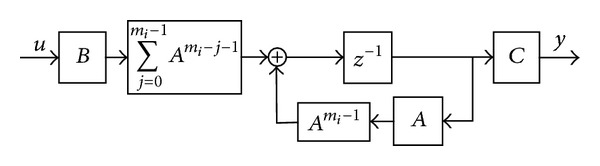
The uncertainty representation of variable sampling rate systems.

Equations (4) then describe the variable sampling rate system of interest. It is interesting to note that the representation for the input matrix is a very neat term of the form ∑_*j*=0_^*m*_*i*_−1^ *A* ^*m*_*i*_−*j*−1^ *B*. One can treat the power of *A*'s as an uncertainty term. Then, the system block diagram representation could be shown in Figure 2. 

Note again that *m*_*i*_ is the number of underlying samples between two consecutive feedback measurements. This is basically an unknown value depending on when the next feedback measurement is available. In the case of BLDC motor driver, the number of samples between measurements depends on the speed of the motor. When the motor is still, *m*_*i*_ may be infinite, but the number should be small when the motor is running at high speed.

## 3. Controller Design

The system in Figure 2 is treated as a nominal system under the influence of plant uncertainties, and the terms *A* ^*m*_*i*_−1^ and ∑_*j*=0_^*m*_*i*_−1^ *A* ^*m*_*i*_−*j*−1^ are the uncertainty terms influencing *A* and *B*, respectively. As discussed before, *A* is the system matrix and is completely known; however, *m*_*i*_ cannot be determined before the next sample measurement is available. Notice that the *A* matrix at this stage is still arbitrary. The authors have not yet discovered any way to further decouple the power of *A*. Therefore, for the moment it seems the best way to treat this variation as a lumped uncertainty. The uncertainty setup is still clearly structured; for the terms *A* ^*m*_*i*_−1^ and ∑_*j*=0_^*m*_*i*_−1^ *A* ^*m*_*i*_−*j*−1^ each affects different part of the system. The structured singular value control synthesis is therefore the best suited design approach to arrive at a less conservative design.

To facilitate the *μ*-synthesis framework, the system uncertainties are represented in their magnitudes:

(5) $$\begin{array}{c} A^{m_{i}-1}=\left(\beta_{A}\Delta_{A}+I\right),\;\text{with}\;\overset{-}{\sigma }\left(\Delta_{A}\right)<1,\\ \sum_{j=0}^{m_{i}-1}A^{m_{i}-j-1}=\left(\beta_{AB}\Delta_{AB}+I\right),\;\text{with}\;\overset{-}{\sigma }\left(\Delta_{AB}\right)<1. \end{array}$$

**Figure 3:**
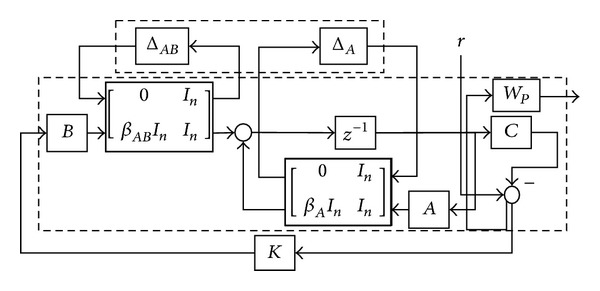
LFT representation of the system.

The control system can be represented with the familiar linear fractional transformation (LFT) as in Figure 3.

The uncertainties Δ_*A*_ and Δ_*AB*_ affect the constant matrices *A* and *B*, respectively. Therefore, there is no need to consider the number of right half plane poles in these cases. The constants *β*_*A*_ and *β*_*AB*_ are scalars to represent the magnitudes of the singular values $\overset{-}{\sigma }\left(A^{m_{i}-1}-I\right)$ and $\overset{-}{\sigma }\left(\sum_{j=0}^{m_{i}-1}A^{m_{i}-j-1}-I\right)$, respectively. Even though the hypothesis allows complete access to *A* and the power of *A*, the maximum singular values of *A* ^*m*_*i*_−1^ still need to be calculated for each *m*_*i*_ to determine the worst case singular values. The same is true for ∑_*j*=0_^*m*_*i*_−1^ *A* ^*m*_*i*_−*j*−1^, and the calculation has to be carried out for every *m*_*i*_. Notice that the structure and the variation of the uncertainties are quite certain in this case. One needs only a step by step multiplication to understand all the powers of *A*, the possible uncertainty and the worst case uncertainty. *W*_*P*_ in Figure 3 allows a reasonable convergence criterion for the synthesis procedure, and it also provides access to the system performance specifications. The setup can then be programmed with the MATLAB toolbox for control synthesis.

## 4. System Description

**Figure 4:**
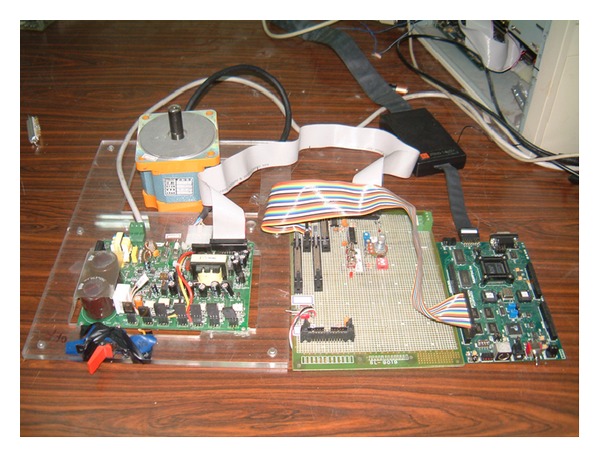
The BLDC system setup.

This experiment uses a BLDC motor from Troy Co., Taiwan, and the TMS320F243 from Texas Instrument for the controller implementation. The driver uses a simple protection circuit to read the hall sensor signal and determine the rotor angle and the rotor speed. Another protection circuit is used to drive the six MOSFET switches for the three-phase winding. The controller determines the proper on-off sequence and the controlled PWM duty cycle for the MOSFET to achieve the control purpose. The schematics of the control circuit and the physical setup are shown in Figure 4.

The capture unit built in the TMS320F243 is used to detect the hall sensor signals and determine the rotor angle. The proposed driver also uses these signals to determine the proper MOSFET to turn it on. There are shunt resistors for constant rate sampling of the phase currents, but the motor speed measurement still depends on the hall sensor signals. The time interval between two consecutive hall sensor signals is used to determine the rotor speed. The sampling rate of the feedback system is thus speed dependent. In this experiment, the underlying sampling frequency for the TMS320F243 is set at 4 KHz. And the PWM module built in TMS320F243 is set to generate PWM waveform whose carrier is also set at 4 KHz. In an other site, there are 12 hall sensor signal updates per revolution for a 4-pole permanent magnet arrangement. Therefore there would be 66 samples between measurements when the motor runs at 3000 rpm.

**Figure 5:**
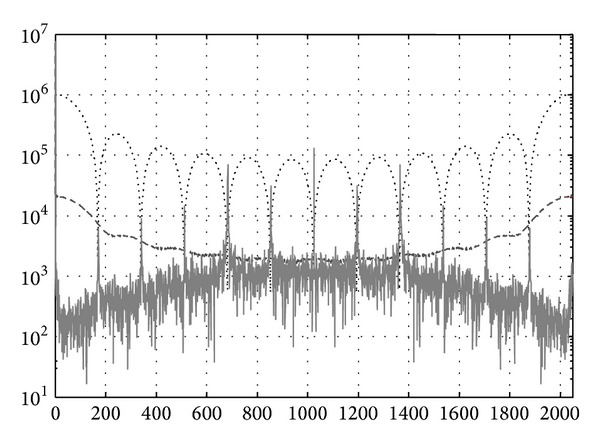
The frequency spectrum of the system response.

Figure 5 shows the frequency spectrum of the open-loop system response. Because the measurement is time varying, the horizontal axis of the figure is a modified frequency unit based on the samples. The signal contains high frequency harmonics over the multiple of twelve. The reason for the noise comes from the mechanical tolerance of the three-hall sensor position. The misalignment of the poles in permanent magnet rotor and the hall sensors induces measurement harmonics with period of 12 samples. Figure 5 also shows the spectrum response (dotted line) of a filter that eliminates signal with period of 12 samples. The filtered signal spectrum (dashed line) indicates that the filter is effective in attenuating the periodic noise. 

The filtered signal can serve as the bases for system identification and for the control feedback. The identified system transfer function is

(6) $$\begin{array}{c} G\left(s\right)=\frac{3.6244}{0.244s+1}. \end{array}$$

Again, there are 12 measurement updates per cycle. For the motor operating at 300~3000 rpm, the sampling frequency would have range of 60~600 Hz. The sampling frequency for the underlying control is 4 KHz. Therefore the number of sampling periods, *m*_*i*_, between feedback measurements would range from 11 to 66. Now it is necessary to calculate the singular values of the powers of $\overset{-}{\sigma }\left(A^{m_{i}-1}-I\right)$ and $\overset{-}{\sigma }\left(\sum_{j=0}^{m_{i}-1}A^{m_{i}-j-1}-I\right)$ for *m*_*i*_ = 11~66 and to obtain the expressions for *β*_*A*_ and *β*_*AB*_. 

**Figure 6:**
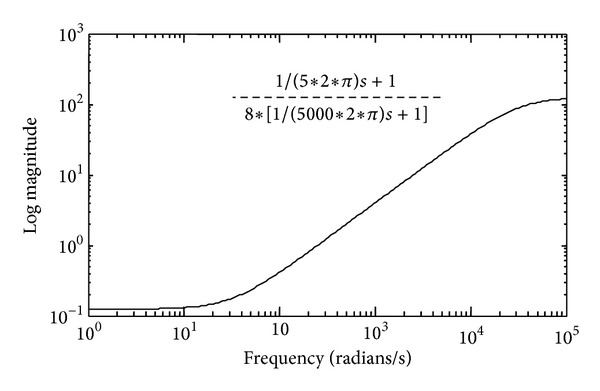
The performance specification for the control synthesis.

The experiment then uses the MATLAB *μ*-synthesis tool box for the control synthesis. The simulation also uses the bilinear transformation to convert the discrete-time system for continuous-time simulation environment. The performance bound is set to the transfer function in Figure 6 for small control error in the low frequency region over the range of sampling rates, to ensure similar performance over a definite range of operating frequencies. It is also interesting to note that the performance bound also helps the control synthesis process to converge. The authors had a hard time making the *μ* value come below 1 before the bound is imposed. The iteration procedure becomes fairly easy when the bound is imposed.

The synthesis resulted in a 6th order controller with the following form:

(7) GC(z)=(4.225z6−14.45z5+14.25z4+3.978z3−16.27z2+10.48z−2.22) ×(z6−3.504z5+3.652z4+0.614z3 −3.704z2+2.478z−0.5362)−1.

**Figure 7:**
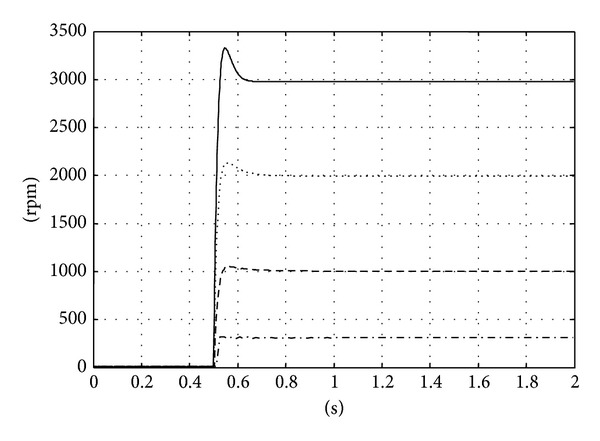
The theoretical system response to the different reference commands.

Figure 7 shows the theoretical responses of the synthesis result under speed command from 300 rpm to 3000 rpm. From the performance setting, the system should have similar responses over the specified speed range. Notice that we have variable sampling rate systems; therefore the number on the time axis is translated from the underlying sampling frequency. One can observe similar responses from the system even when the different setting actually changes the system behavior.

**Figure 8:**
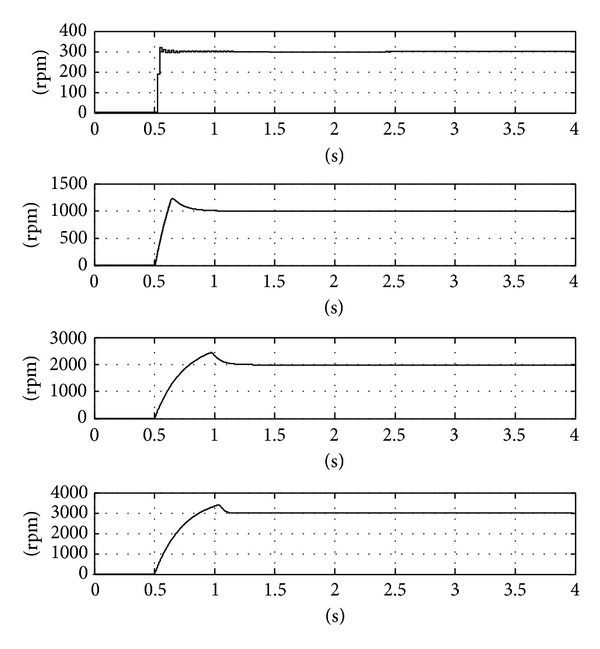
Simulation response of the system under different reference commands.

The controller calculates a PWM duty cycle in the actual system. Inherently, there is no negative command from the duty cycle. There is a separate logic for the reversed rotation, but it will not be discussed here. The lack of negative command would result in slower recovery from the overshoot. The MOSFET switches also impose limits on the output current.  Figure 8 shows the simulation responses. The traces in the figure show the responses to 300, 1000, 2000, and 3000 rpm command. Due to the saturation and the nonnegative control output effect, the responses are slower and the recovery from the overshoot is comparatively slow. However, the tracking performances become similar once the responses come close to the set points.

## 5. Controller Implementation

The TMS320F243 is a 16-bit fixed-point digital signal processor. There are special considerations when implementing high order control algorithm. The compensator is first decomposed into fractional expansion in Figure 13, where one of the second order terms has been omitted because it has a gain close to zero.

**Figure 9:**
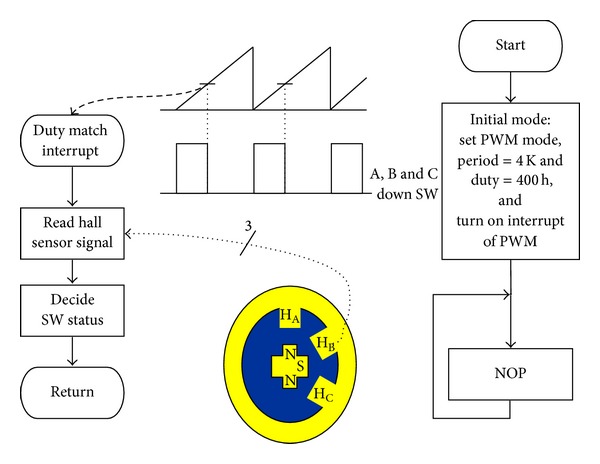
The hard implementation and the controller flow chart.

The first two terms in Figure 13 use Q15 and Q14 formats, respectively, to avoid truncation error. The direct gain term is represented in the less detailed Q13 format because there is no recursive calculation. The inverter determines the new set of MOSFET switches to turn off and turn on, and the controller will compute the speed measurement and update the control law. The new control duty cycle takes effect when the counter enters the counting for the next sampling period.  Figure 9 illustrates the working process of the firmware.

## 6. Experimental Results

**Figure 10:**
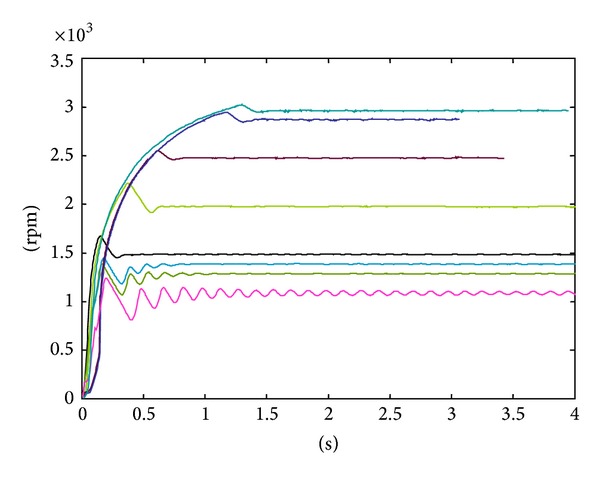
The experimental response of system under different reference commands.

**Figure 11:**
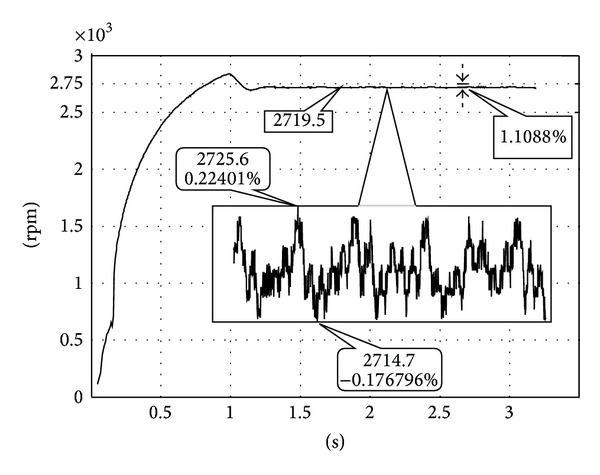
Detailed response.

**Figure 12:**
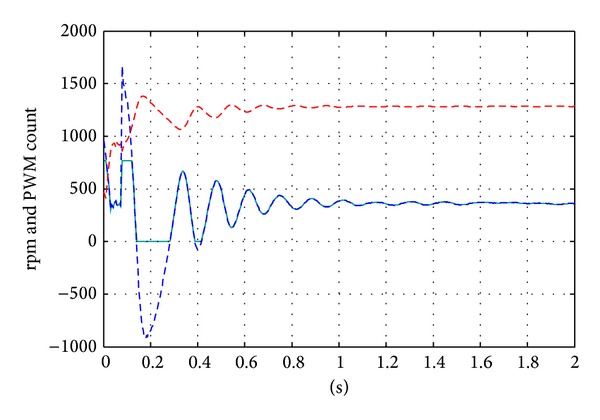
Saturation effect and the effect of nonnegative efforts.

**Figure 13:**
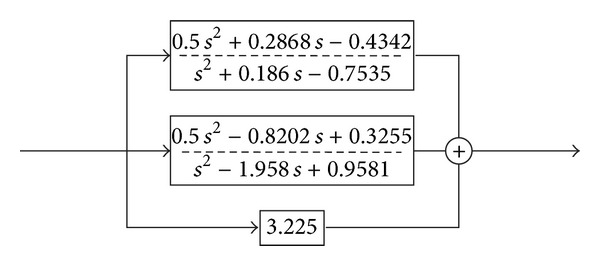
The fractional expansion of the controller.

Figure 10 shows the results of the control when the reference speed varies from 1000 to 3000 rpm. The experiments only carry the responses down to 1000 rpm because negative control efforts start appearing, and the PWM setup has difficulty representing negative efforts. From Figure 10, one observes that the controller achieves similar system responses when the speed commands are around the high speed range. The slow rising times in the initial transient are the result from driver saturation. The effect is included in the simulation but is not included in the controller synthesis. The responses grow oscillatory as the speed command is reduced to close to 1000 rpm (near the limit of the uncertainty assumptions.) A closer look at the responses (Figure 11) shows that the controller achieves very good 1% steady state error performances over the range of speed commands. The speed variation which is also very important in precision applications remains within a range less than ±0.3%. This is considered a very accurate servo performance in the precision engineering applications. As mentioned before, the PWM drive signal does not reflect negative control efforts.  Figure 12 illustrates the effect of nonnegative control efforts and also the effect of control saturations. It is easy to see that the control saturation significantly prolonged the system response time. The nonnegative control output not only prolongs settling time but also adds to the oscillation. As mentioned before, this effect reduced the performance of the proposed controller in low speed operation. However, the experiments still demonstrated the effect of the control over the specified speed range. 

## 7. Conclusion

This paper proposed a controller design for systems with variable sampling rate. By variable sampling rate, the authors mean a system with undetermined sampling frequency or speed dependent sampling frequency. The paper first presented the system modeling for the variable sampling rate system. The changing sampling rate was then translated into system uncertainties. The uncertainties appear in different locations in the system with fixed structure; therefore, the popular *μ*-synthesis procedure is introduced to calculate the controller. The experimental verification is based on the TMS320F243 digital signal processor. The experimental results over different speed settings agree with the original design specifications and have demonstrated the effectiveness of the proposed method.
